# The effect of smartphone addiction on the relationship between psychological stress reaction and bedtime procrastination in young adults during the COVID-19 pandemic

**DOI:** 10.1186/s12888-023-05276-9

**Published:** 2023-11-07

**Authors:** Zhenliang Yang, Jiahao Huang, Ziqi Li, Hui Xu, Chenguang Guo

**Affiliations:** 1 Department of Medical Imaging, The First Affiliated Hospital of Xi’an Jiaotong University, Xi’an, 710061 China; 2 Faculty of Psychology, Tianjin Normal University, Tianjin, 300387 China; 3 School of Mental Health, Wenzhou Medical University, Wenzhou, 325035 China

**Keywords:** Psychological stress reaction, Smartphone addiction, Family cohesion, Bedtime procrastination, Young adults

## Abstract

**Background:**

Previous studies on bedtime procrastination mainly focused on the influencing factors of stress and draw less attention on the role of family environment.

**Aim:**

This study aimed to explore the effect of psychological stress reaction on bedtime procrastination in young adults, with considering the mediating effect of smartphone addiction, and the moderating effect of family cohesion during the COVID-19 pandemic.

**Methods:**

A sample of 1217 young adults completed psychological stress reaction scale, Smartphone addiction tendency scale for young adults, bedtime procrastination scale and family cohesion scale. A moderated mediation model was conducted to clarify the effect of psychological stress reaction on bad bedtime procrastination in young adults.

**Results:**

The findings showed that: (1) The individual level of psychological stress reaction was positively associated with bedtime procrastination; (2) Smartphone addiction mediated the effect of psychological stress reaction on bedtime procrastination; (3) Family cohesion moderated the relationship among psychological stress reaction, smartphone addiction and bedtime procrastination.

**Conclusions:**

This study revealed the effect of smartphone addiction on the relationship between psychological stress reaction and bedtime procrastination during the COVID-19 pandemic, and these findings could provide novel evidence that family cohesion may serve as a protective factor against the negative consequences of smartphone addiction on bad bedtime procrastination.

**Supplementary Information:**

The online version contains supplementary material available at 10.1186/s12888-023-05276-9.

## Introduction

One third of life is spent in sleep. Adequate and good sleep is the basis for normal learning, living, adapting to the environment and ensuring physical and mental health [1]. However, the current sleep status of young adults is not optimistic, that more young adults have sleep disorders, especially the lack of sleep time and the procrastination in falling asleep time [2]. The general lack of sleep time is mainly due to the procrastination in falling asleep [3], while young adults with a tendency to sleep late often show significant delays in daytime activities and academic completion [4]. This shows that procrastination is likely to play an important role in the performance of healthy sleep behaviors. The Dutch scholar Kroese introduced bedtime procrastination into the field of procrastination and proposed that bedtime procrastination refers to the behavior that an individual cannot go to bed at a predetermined time without being hindered by external factors [5, 6]. Previous study shows that bedtime procrastination has a negative impact on sleep quality, and in the long run it would cause irreversible damage to individual physical and mental health [7]. Therefore, this study aims to provide constructive suggestions and theoretical basis for the intervention of bedtime procrastination and the improvement of physical and mental health by exploring the psychological factors and mechanisms that affect bedtime procrastination.

In December 2019, COVID-19 appeared in Wuhan, Hubei Province for the first time and quickly spread to 24 countries across the country and the world [8]. On January 30, 2020, the World Health Organization declared the COVID-19 as a public health emergency of national concern. This pandemic is widespread, lacking specific drugs, and will endanger lives if not treated in time. Its sudden onset and severity are far more than people’s expectations [9]. In addition to the pandemic itself, the information overload of the pandemic has caused a huge impact and impact on the psychology of the masses. Su Binyuan tracked the characteristics of psychological stress reaction and time course of the people in different stages of the COVID-19 in the past five weeks [10], and found that the anxiety, depression, compulsion, insomnia and other psychological symptoms caused by the pandemic have alleviated, but the overall level of psychological stress is significantly higher than the reference level in the non-pandemic period. This finding suggested that the level of people’s psychological stress reaction generally increased during the COVID-19 pandemic.

In addition, young adults have always been a group that needs attention. They lack in social experience and coping ability and are more vulnerable to the impact of negative environment [11]. The existing literature on the direct relationship between psychological stress reaction and bedtime procrastination in the era of the COVID-19 pandemic is still less, and the internal mechanism of the impact of psychological stress reaction on bedtime procrastination also needs further exploration. Based on this, the purpose of this study is to explore the effect of psychological stress reaction on bedtime procrastination and its internal mechanisms in young adults during the COVID-19 pandemic.

### Relationship between psychological stress reaction and bedtime procrastination

**Fig. 1:**
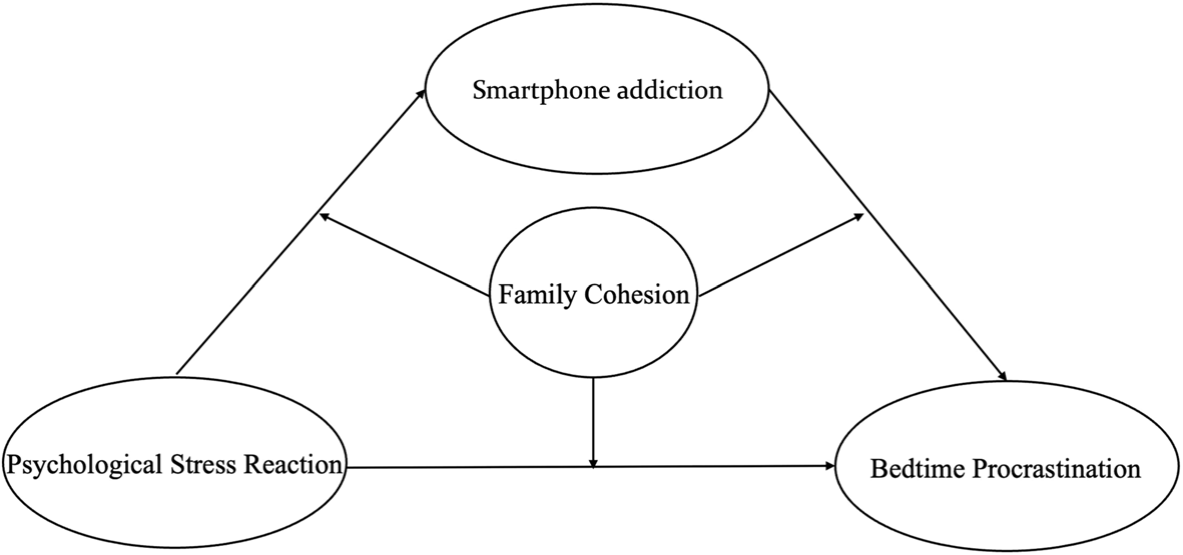
The moderated mediation theory model in this study

In the current era of COVID-19 pandemic, stressors are everywhere, and these multiple stressors also have a huge impact on mental health and sleep [12, 13]. Some studies have shown that feeling stress is the main obstacle to sleep. When exposed to unpredictable or uncontrollable stressors, sleep will be affected [14]. According to the theory of self-consumption, long-term exposure to stress will have a negative impact on the individual’s self- regulatory ability, leading to procrastination [15]. Bedtime procrastination is a kind of procrastination related to sleep. During the COVID-19 pandemic, a survey showed that 89.8% of the participants went to bed after 10 p.m. on weekdays, compared with 57.1% before the pandemic [16]. In another survey, the participants slept an hour and 13 min late on average every day during the COVID-19 pandemic [17]. Compared with insomnia and other sleep disorders that lead to insufficient sleep, bedtime procrastination is more common in the general population, which means that understanding its underlying mechanism may provide new ideas and ways to solve the problem of insufficient sleep in the general population [18]. Therefore, we will test our hypothesis 1: the psychological stress reaction of young adults can predict bedtime procrastination during the COVID-19 pandemic (Fig. 1).

### Mediating role of Smartphone addiction

Smartphone addiction is defined as the psychological or behavioral problems of Smartphone users caused by the abuse of Smartphones [19, 20]. According to the general strain theory, problem behaviors are mainly caused by negative experiences brought about by various stresses. Stress is the specific factor of substance addiction and relapse of addictive behavior [21]. In the study of internet addiction and Smartphone addiction, it is a kind of technology addiction [22]. When individuals feel internal and external stress, they will use the internet and Smartphone excessively in order to distract from the stress [23]. Young believes that the behavior of Internet addicts can be seen as a measure to reduce perceived stress [24]. Smartphone addiction can also be seen as a way to release daily pain and tension. Previous studies showed that stress can significantly predict Smartphone addiction [25]. In addition, Smartphone addiction can lead to bedtime procrastination [26]. According to the Internet satisfaction theory, people can seek satisfaction by increasing the time they spend using smartphones [27]. Nowadays, the pace of modern life is fast. People who under the stress of work, study and family during the daytime want to make full use of the time before bed to meet their psychological needs. Activities such as chasing dramas and playing games are easy to immerse people in them without knowing, which may lead to bedtime procrastination [28, 29]. Other researchers believe that using electronic devices such as Smartphones before going to bed will have a negative impact on the sleep rhythm of teenagers, and the blue light of electronic screens will affect the normal secretion of melatonin, which is also an important reason for young adults to sleep more and more late [30]. Based on this, this study proposes the hypothesis 2: Smartphone addiction plays a mediating role in the relationship between psychological stress reaction and bedtime procrastination (Fig. 1).

### Family cohesion moderation

In order to stop the spread of the COVID-19 to the campus, the Ministry of Education requested to postpone the start of school in the spring of 2020. In the spring of 2022, some colleges and universities were also procrastinated due to the pandemic. For young adults, extended vacations, long-term home stay, less going out, and unable to go to school to study and socialize normally may affect their studies and aggravate psychological stress reactions such as anxiety and depression [31–33]. The theory of interaction between individuals and environment points out that individual behavior problems are the result of interaction between individual factors and environmental factors [34]. Studies have shown that there is a significant negative correlation between family cohesion and Smartphone addiction. Liu Shihong defined family cohesion as the degree of emotional connection with family members that individuals feel. The better the family atmosphere [35], the more communication, the less abnormal behavior individuals will have [36]. In terms of environmental factors, the family is one of the micro systems that directly affect the psychological development of young people. Compared with other family variables (such as family upbringing, parent–child communication), family cohesion can better measure the overall atmosphere of the family and is a comprehensive indicator that reflects the positive family atmosphere and the close relationship between family members [37]. Family environment factors, including the attitude of raising children, family communication and cohesion, can protect the excessive use of the Internet and addictive behavior [38]. Other researchers found that family environmental factors, such as family structure, parents’ behavior, and family socio-economic status, can affect individual sleep quality and sleep time in various ways [39]. Missildine proposed that the “procrastination syndrome” was caused by parenting styles, including over forcing children and setting unrealistic goals [40]. When children cannot meet their parents’ expectations, they will begin to feel anxious and unworthy of doing, which will lead to procrastination [41]. Aggressive parenting styles such as corporal punishment in parenting styles have a significant negative correlation with family cohesion [42]. During the pandemic period, young adults procrastinated their school start and stayed at home longer. Based on this, this study proposes the hypothesis3: family cohesion moderates the relationship between psychological stress behavior and Smartphone addiction, psychological stress behavior and bedtime procrastination, as well as Smartphone addiction and bedtime procrastination (Fig. 1).

## Methods

### Participants and procedures

Participants were college students recruited online to answer the study questionnaire. Most of them were from two university in the City of Shenyang and Tianjin, where the frst author attended undergraduate and postgraduate program and advertised the study on campus. Some participants were from other universities in China. The recruiters are all postgraduate students majoring in psychology. In total, 1241 young adults were included in an online questionnaire survey using the convenient sampling method in September 2022. After eliminating invalid questionnaires, 1217 valid questionnaires were collected, with an effective rate of 98.07%, including 698 males (57.35%) and 519 females (42.65%). All the measures administered in Chinese.

### Measures

Psychological Stress Reaction Scale: the Chinese version of SRQ-20 scale [43] was adopted, with a total of 20 items (for example, “Do you often have headaches?”, “Do you feel unhappy?”), Each item is scored at 2 points, with 0 indicating “No” and 1 indicating “Yes”. This scale mainly measures individual psychological stress reactions such as anxiety and depression. The total score of each item is the total score of psychological stress reaction, and the higher the score is, the higher the level of psychological stress reaction is. Cronbach’s alpha coefficient of this scale in this study is 0.922.

Smartphone addiction tendency scale for young adults: the Smartphone addiction tendency scale for young adults [44] was adopted, A total of 16 items are included (for example: “I would feel lonely without a Smartphone”, “I would rather chat on a Smartphone than communicate directly face to face”). Each item is scored at 5 points. 1 means “very inconsistent”, and 5 means “very consistent”. The sum of the scores of each item is the total score of Smartphone addiction. The higher the total score, the higher the degree of individual Smartphone addiction. There are four factors in total, including withdrawal symptoms, salient behavior, social comfort and mood change. Cronbach’s alpha coefficient of this scale in this study is 0.951. The fitting indexes of the confirmatory factor analysis model in this study are: CFI = 0.930, TLI = 0.914, RMSEA = 0.091, SRMR = 0.039, indicating that the scale has good structural validity.

Bedtime procrastination Scale: the bedtime procrastination Behavior Scale for young adults revised by Ma Xiaohan [45], which consists of nine items (such as “I don’t go to bed on time”), is scored with 5 points, with 1 representing “never” and 5 representing “always”, and items 2, 3, 7 and 9 are scored in reverse. The average score of all items is the scale score. The scale score ranges from 1 to 5. The higher the score is, the more serious the bedtime procrastination behavior of the individual is. In this study, Cronbach’s alpha coefficient of this scale is 0.804.

Family cohesion scale: using the family cohesion scale compiled by Olson and revised by Fei Lipeng [46], there are 16 questions in total (for example, “when there are difficulties, family members will try their best to support each other”), and 5 points are used, with 1 indicating “no” and 5 indicating “always”. The higher the score, the better the family cohesion. In this study, Cronbach’s alpha coefficient is 0.896.

### Statistical analysis

The unified questionnaire was used for the test. In the instruction, the participants were required to answer carefully according to the actual situation, and the confidentiality of personal information was emphasized. SPSS 26.0 is used to input and manage the collected data, and descriptive statistical analysis and correlation analysis are conducted. After the scores of each scale were standardized, two models were performed in the Process macro program [47]: model 4 was used to test the mediation of Smartphone addiction, and model 59 was used to test the mediation of family cohesion. After Bonferroni correction, the threshold value of *P* < 0.05 was considered statistically significant. R studio was used for confirmatory factor analysis.

## Results

### Inspection and control of common method bias

Since all variables in this study were collected by self-reported method, the results may be affected by common method bias. According to the suggestions of Podsakoff and his colleagues [48], the procedures have been controlled accordingly, such as protecting the anonymity of the responders and reducing the degree of speculation about the measurement purpose; In order to further improve the preciseness of the study, this study used Harman’s single factor test to test the common method deviation. The results showed that there were 8 factors with eigenvalues greater than 1, and the variance interpretation rate of the first factor was 20.80%, less than the critical value of 40% [48], indicating that there was no serious common method bias in the data of this study.

### Descriptive statistics and correlation analysis

**Fig. 2:**
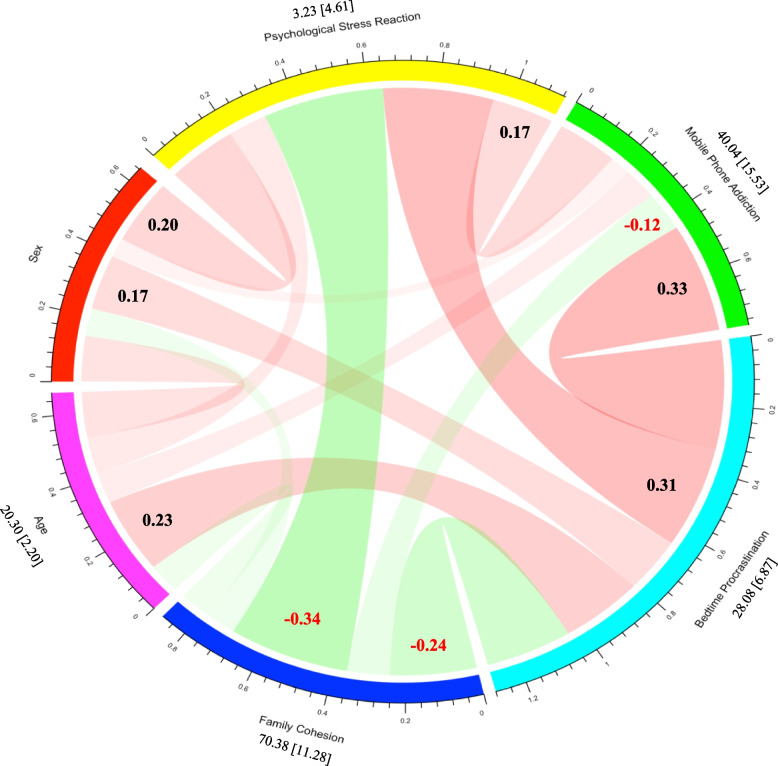
The relationship between all variables in this study. Black and red numbers represent significantly (*p* < 0.05) positive and negative effects, respectively. The numbers above variable names represent Mean value [Standard deviation]

The mean value, standard deviation and Pearson correlation analysis results of each variable are shown in Fig. 2. Psychological stress reaction was positively correlated with Smartphone addiction (*r* = 0.17, *p* < 0.01) and bedtime procrastination (*r* = 0.31, *p* < 0.01); There was a significant positive correlation between Smartphone addiction and bedtime procrastination (*r* = 0.33, *p* < 0.01); Family cohesion was negatively correlated with psychological stress (*r* = -0.34, *p* < 0.01), Smartphone addiction (*r* = -0.12, *p* < 0.01) and bedtime procrastination (*r* = -0.24, *p* < 0.01). In addition, the correlation between gender, age and main research variables showed that gender was significantly related to psychological stress (*r* = 0.20, *p* < 0.01), Smartphone addiction (*r* = 0.06, *p* < 0.01), bedtime procrastination (*r* = 0.17, *p* < 0.01) and family cohesion (*r* = -0.08,* p* < 0.01), while age was significantly related to psychological stress (*r* = 0.11, *p* < 0.01), Smartphone addiction (*r* = 0.10, *p* < 0.01) Bedtime procrastination (*r* = 0.23, *p* < 0.01) was significantly correlated with family cohesion (*r* = -0.90, *p* < 0.01) (Fig. 2).

### Mediation effect analysis of Smartphone addiction

Model 4 in the SPSS macro program PROCESS developed by Hayes [47] was used to test the mediating role of Smartphone addiction between psychological stress reaction and bedtime procrastination after controlling gender and age. The results show that (Table 1): psychological stress reaction habits can significantly and positively predict Smartphone addiction (*β* = 0.161, *p* < 0.001) and bedtime procrastination (*β* = 0.227, *p* < 0.001), Smartphone addiction can significantly positively predict bedtime procrastination (*β* = 0.272, *p* < 0.001). The intermediary effect analysis shows that the total effect of smartphone addiction is 0.27, the direct effect is 0.23, and the indirect effect is 0.04, the 95% confidence interval of Bootstrap is [0.218, 0.324], [0.175, 0.279],[0.027, 0.062] respectively, and the confidence interval does not include 0, indicating that the intermediary effect of bedtime procrastination is significant, accounting for 16.236% of the total effect.

**Table 1:** An analysis of the mediating effect of Smartphone addiction

Mediator	Effect	Effect value	Boot Standard error	95% Confidence interval
Smartphone addiction	Total effect	0.271	0.027	[0.218, 0.324]
	Direct effect	0.227	0.026	[0.175, 0.279]
	Indirect effect	0.044	0.009	[0.027, 0.062]

### Analysis of moderating effects of family cohesion

Model 59 in the PROCESS program was used to test the moderating effect of family cohesion after controlling gender and age. During the test, the percentile Bootstrap method with deviation correction was used to determine the significance of the moderating effect. The results are shown in Table 2: Psychological stress reaction (*β* = 0.213, *p* < 0.001), family cohesion (*β* = -0.071, *p* < 0.05) can significantly predict Smartphone addiction, and the interaction between psychological stress reaction and family cohesion can significantly predict Smartphone addiction (*β* = 0.142, *p* < 0.001), indicating that family cohesion can moderate the relationship between psychological stress reaction and Smartphone addiction; Psychological stress reaction can positively predict bedtime procrastination (*β* = 0.225, *p* < 0.001), significant negative bedtime procrastination in family cohesion (*β* = -0.108, *p* < 0.001), the interaction between psychological stress reaction and family cohesion can positively predict bedtime procrastination (*β* = 0.061, *p* < 0.05), indicating that family cohesion can significantly adjust the prediction of psychological stress reaction on bedtime procrastination; In addition, Smartphone addiction has a significant positive predictive effect on bedtime procrastination (*β* = 0.231, *p* < 0.001), and the interaction between Smartphone addiction and family cohesion also has a significant predictive effect on bedtime procrastination (*β* = 0.088, *p* < 0.01), that is, the relationship between Smartphone addiction and bedtime procrastination is moderated by family cohesion.

**Table 2:** Bias of mediating moderating effect of psychological stress reaction on bedtime procrastination

Variables	Model 1: (Calibration: Smartphone addiction)			Model 2: (Calibration: bedtime procrastination)		
	*β*	*SE*	*t*	*β*	*SE*	*t*
Age	0.030	0.013	2.283*	0.071	0.012	6.101***
Sex	0.019	0.058	0.321	0.168	0.052	3.213**
Psychological Stress Reaction	0.213	0.033	6.433***	0.225	0.030	7.403***
Family Cohesion	-0.071	0.030	-2.391*	-0.108	0.028	-3.895***
Psychological Stress Reaction × Family Cohesion	0.142	0.026	5.357***	0.061	0.025	2.467*
Smartphone addiction				0.231	0.027	8.662***
Smartphone addiction × Family Cohesion				0.088	0.029	3.067**
*R*^*2*^	0.062			0.239		
*F*	15.945***			54.332***		

*β* Standardized partial regression coefficient, *SE* Standardized standard error

^*^: *p* < .05; **: *p* < .01; ***: *p* < .001

**Fig. 3:**
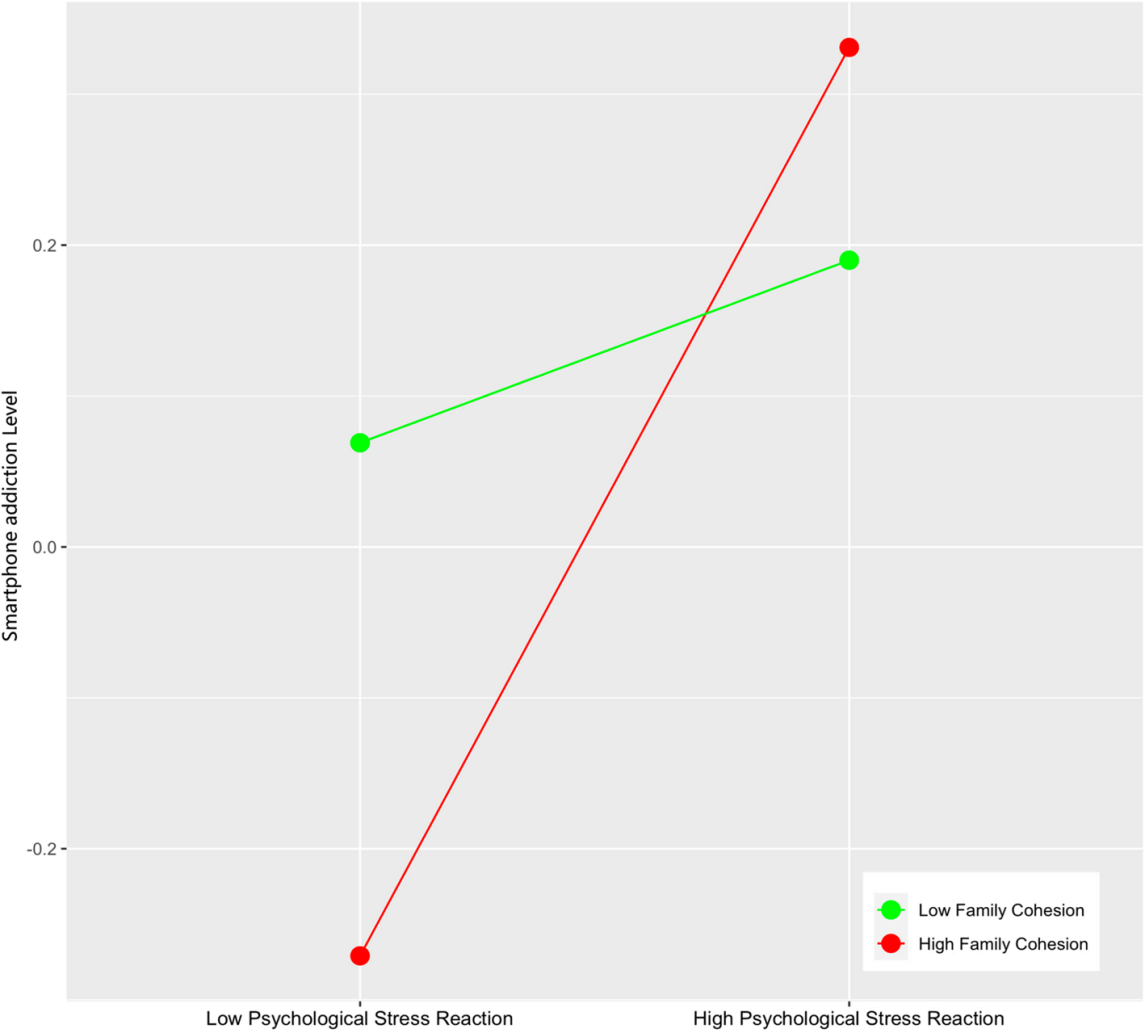
The Moderating Effect of Family Cohesion on Psychological Stress Reaction and Smartphone addiction

**Fig. 4:**
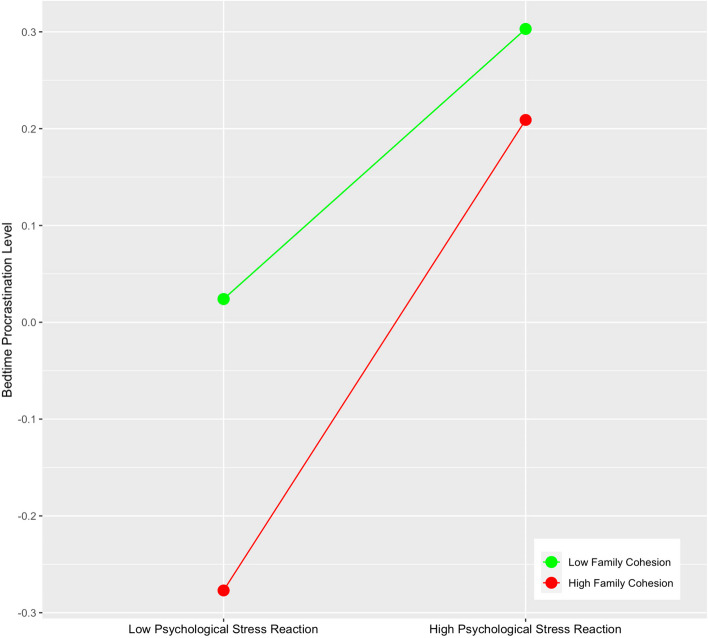
The Moderating Effect of Family Cohesion on Psychological Stress Reaction and Bedtime Procrastination

**Fig. 5:**
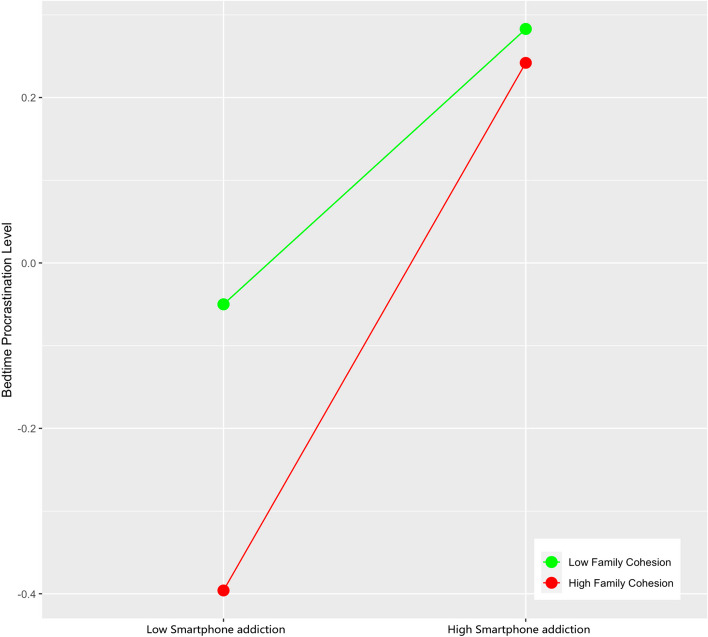
The Moderating Effect of Family Cohesion on Smartphone addiction and Bedtime Procrastination

In order to more clearly show the moderating role of family cohesion, this study further conducted a simple slope test and drew a simple effect analysis chart. The results showed that when the individual’s family cohesion was low, the psychological stress reaction had a significant positive predictive effect on Smartphone addiction (β Simple = 0.071, *t* = 2.178, *p* < 0.05), when family cohesion is high, the positive predictive effect of psychological stress reaction on Smartphone addiction is enhanced (β simple = 0.354, *t* = 7.065, *p* < 0.001) (Fig. 3). The simple effect analysis of family cohesion on moderating psychological stress reaction and bedtime procrastination shows that when the individual’s family cohesion is low, bedtime procrastination shows a significant upward trend with the increase of psychological stress reaction scores (β Simple = 0.164, *t* = 5.493, *p* < 0.001), when family cohesion is high, the positive predictive effect of psychological stress reaction on bedtime procrastination is enhanced (β simple = 0.286, *t* = 6.1233, *p* < 0.001) (Fig. 4). The simple effect analysis of family cohesion on the moderating of Smartphone addiction and bedtime procrastination shows that with the improvement of individual family cohesion, the predictive role of Smartphone addiction on bedtime procrastination is gradually enhanced (from β Simple = 0.144, *t* = 3.292, *p* < 0.01 enhanced to β Simple = 0.319, *t* = 9.381, *p* < 0.001) (Fig. 5).

## Discussion

This study provides a clearer understanding of the role of family cohesion in understanding the relationship between bad bedtime procrastination and two important behavioral and mental outcomes: psychological stress reaction and Smartphone addiction. The results show that psychological stress reaction can not only directly predict individual bedtime procrastination behavior, but also indirectly affect bedtime procrastination through the intermediary variable of Smartphone addiction. Furthermore, family cohesion moderates this influence. This suggests that family cohesion may be an important target for interventions focused on trying to ameliorate the effects of psychological stress reaction on bad bedtime procrastination in young adults.

### Relationship between psychological stress reaction and bedtime procrastination

This study found that under the pandemic situation, the psychological stress reaction was significantly positively correlated with bedtime procrastination, that is, the higher the level of psychological stress reaction of young adults, the more obvious their bedtime procrastination behavior was, which was consistent with the correlation between stress and sleep problems in previous studies [17]. Previous studies have shown that the level of individual psychological stress reaction significantly increased during the COVID-19 pandemic [10], and the COVID-19 pandemic has a great impact on individual sleep behavior [17]. In the stress health model, psychological stress will have a negative impact on sleep, higher psychological stress will lead to poor sleep quality [49], and poor sleep quality will lead to increased bedtime procrastination, which support the theory of self-regulatory resources [18]. Young adults may consume excessive self- regulatory resources during the daytime due to COVID-19 related events, and the use of resources will be temporarily exhausted, leaving the body in a state of self-depletion [50]. Therefore, the self- regulatory resources that can resist late sleep at night appear insufficient, resulting in self-regulation failure and bedtime procrastination [18]. However, a large number of studies have proved that bedtime procrastination can directly or indirectly lead to immune system disorder, increasing the risk of people suffering from cancer, diabetes, obesity, chronic infection and other diseases [51, 52]. Staying up late has caused negative effects on the physical and mental health of contemporary people that cannot be ignored, such as staying up late is easy to induce sudden death, increase the risk of inducing metabolic syndrome, and easily lead to memory decline [7]. Therefore, the results of this study indicate that the psychological stress reaction during the pandemic period is an important risk factor affecting bedtime procrastination of young adults, which suggest that great attention should been attached to the psychological stress reaction of young adults during the pandemic period to reduce bedtime procrastination.

### Mediation of bedtime procrastination

This study showed that Smartphone addiction played a part of intermediary role between psychological stress reaction and bedtime procrastination, indicating that psychological stress reaction could not only directly affect bedtime procrastination behavior, but also indirectly affect bedtime procrastination through Smartphone addiction. Previous studies have confirmed that there is a close relationship between psychological stress reaction and Smartphone addiction, and the Internet environment or using Smartphones can temporarily escape unpleasant experiences and stresses in the real world. However, compulsive use of Smartphones to obtain satisfaction and happiness may eventually lead to addiction to Smartphones [53]. Previous research based on the general strain theory found that stress can significantly predict Smartphone addiction, and excessive use of Smartphones may become a way to release the daily pain and tension. The research of Lung [54] shows that the unprecedented virus outbreak will cause great stress on the public of different ages, regions and occupations. The COVID-19 pandemic has increased the public’s psychological stress reaction, which will become the reason for increasing Smartphone addiction. In addition, the results of this study on Smartphone addiction and bedtime procrastination are also consistent with those of predecessors [26]. Just like the Internet satisfaction theory, the use of Smartphones will increase individual satisfaction [27]. Due to the increased psychological stress caused by the pandemic, individuals will inevitably use Smartphones to meet their psychological needs before going to bed. Immersive use of electronic media before sleep is also more likely to make individuals lose sense of time and delay sleep [55]. In conclusion, the results of this study suggest that we should actively pay attention to young adults’ Smartphone addiction behavior in our daily life, especially when the psychological stress reaction is relatively strong during the pandemic, and take some intervention measures when necessary, such as more aerobic exercise and more paper reading [56], to reduce the Smartphone addiction tendency and thus reduce the negative impact of bedtime procrastination.

### Moderating effects of family cohesion

More importantly, our study found that family cohesion can play a moderating role in the direct and indirect path between psychological stress reaction and bedtime procrastination. Specifically, family cohesion can significantly adjust the impact of psychological stress reaction on bedtime procrastination, that is, compared with young adults with low family cohesion, psychological stress reaction has a stronger predictive effect on bedtime procrastination of young adults with high family cohesion. In addition, family cohesion can also moderate the mediating effect of Smartphone addiction (including the first half and the second half of the path), that is, the relationship between psychological stress reaction and Smartphone addiction, as well as the relationship between Smartphone addiction and young adults’ bedtime procrastination, are both moderated by family cohesion. Our results suggest that a good family environment is a protective factor for bedtime procrastination, that is, individuals with a good family environment tend to have less Smartphone addiction and bad bedtime procrastination, which is consistent with the conclusions of existing literature [38, 39]. However, when the interaction between family density and psychological stress reaction is used to predict Smartphone addiction, the results show that individuals with high family cohesion are more likely to have Smartphone addiction and bedtime procrastination. Nowadays, the use of Smartphones is becoming more important in family communication. Smartphone communication not only helps teenagers keep in touch with their parents, but also helps them develop identity and independence. Lin’s research confirms that individual Smartphone communication has a significant correlation with family cohesion [57]. However, excessive use of Smartphones may lead to addiction. We speculate that in our study, individuals with high family cohesion are addicted to Smartphones due to more smartphone communication. The results of the interaction between psychological stress reaction and family cohesion, Smartphone addiction and family cohesion also indicate that individuals with high family cohesion are more likely to have bedtime procrastination behavior. The reason may be that higher family cohesion means better family atmosphere, more communication between family members, parents may treat children more in a warm and understanding way [58], and they respect children’s choices more, This may include their sleep time. Of course, the reason for this result may also be that compared with psychological stress reaction and Smartphone addiction, the protection of family cohesion is not strong, and it is difficult to dominate when interacting with other variables.

## Conclusion

This study shows that the psychological stress reaction during the pandemic is related to the increased bedtime procrastination caused by increasing Smartphone addiction, which is particularly obvious in the group with high family cohesion in young adults. These results highlight a potentially important role for family cohesion in protecting against two behavioral and mental outcomes known to be associated with poor bedtime procrastination: psychological stress reaction and Smartphone addiction. Given that family cohesion could be as a viable target for sleep intervention, further longitudinal innervation studies are needed to prove these inter-relationships more rigorously. In addition, cross-sectional design may not be suitable for mediation analysis, which is also a limitation of this study. We will also use longitudinal design to continue future research.

### Supplementary Information

**Additional file 1:** **Figure S1.** Effect of Psychological stress reaction on Smartphone addiction with Johnson-Neyman confidence bands. **Figure S2.** Effect of Psychological stress reaction on Bedtime procrastination with Johnson-Neyman confidence bands. **Figure S3.** Effect of Smartphone addiction on Bedtime procrastination with Johnson-Neyman confidence bands.

## Data Availability

The data that support the findings of this study are available on request from the corresponding author.
