# Supplementary material for: The effect of smartphone addiction on the relationship between psychological stress reaction and bedtime procrastination in young adults during the COVID-19 pandemic
Source: BMC Psychiatry. 2023 Nov 7;23:813. doi: 10.1186/s12888-023-05276-9 (PMC10631052; doi:10.1186/s12888-023-05276-9)
Supplement: Supplementary file 1 — Additional file 1: Figure S1. Effect of Psychological stress reaction on Smartphone addiction with Johnson-Neyman confidence bands. Figure S2. Effect of Psychological stress reaction on Bedtime procrastination with Johnson-Neyman confidence bands. Figure S3. Effect of Smartphone addiction on Bedtime procrastination with Johnson-Neyman confidence bands. [file 12888_2023_5276_MOESM1_ESM.docx]

Supplementary Materials

In order to understand the critical values of moderating effects and the range of interval values for statistically different moderating variable, this study also used the Johnson Neyman method for a simple slope test [68] to examine the relationship among family cohesion moderating psychological stress reaction, smartphone addiction, and bedtime procrastination, To obtain a significant interval for the simple slope coefficient of the moderating effect. Figure S1 shows that in the moderating of family cohesion on the relationship between psychological stress reaction and smartphone addiction, when the family cohesion level is below -2.267 or above -1.043, the 95% confidence interval of the simple slope does not include 0, and the simple slope is significant, indicating a significant moderating effect; Figure S2 shows that in the moderating of family cohesion on the relationship between psychological stress reaction and bedtime procrastination, when the family cohesion level is below -0.541, the 95% confidence interval of the simple slope does not include 0, and the simple slope is significant, indicating a significant moderating effect. In other words, the impact of psychological stress reaction on bedtime procrastination weakens with the increase of family cohesion level; Figure S3 shows that in the moderating of family cohesion on the relationship between smartphone addiction and bedtime procrastination, when the family cohesion level is higher than -1.430, the 95% confidence interval of the simple slope does not include 0, and the simple slope is significant, indicating a significant moderating effect. In other words, the impact of smartphone addiction on bedtime procrastination increases with the increase of family cohesion level.

**
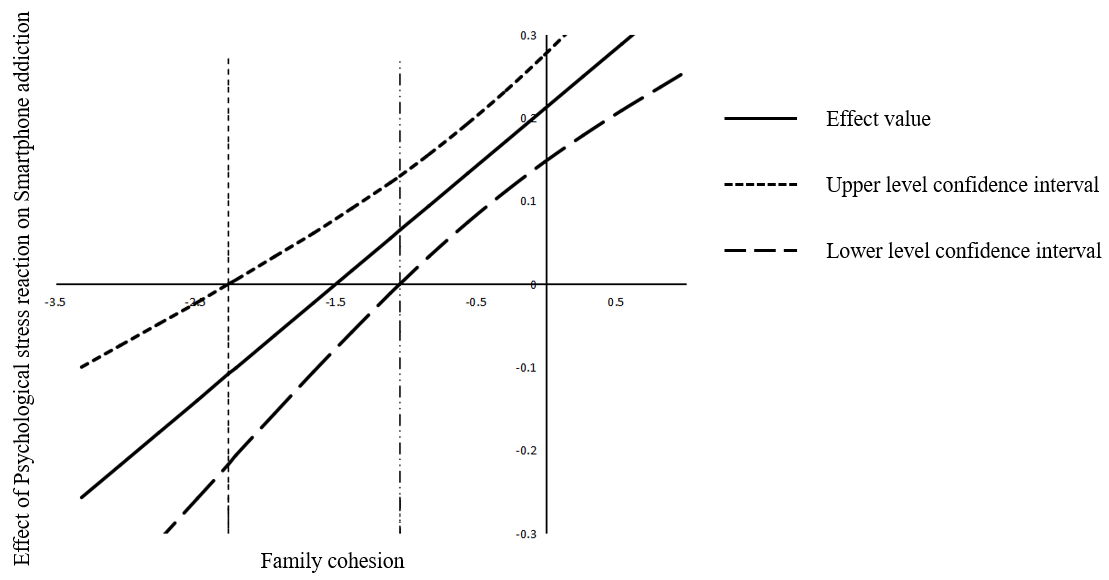
**

**Figure S1.** Effect of Psychological stress reaction on Smartphone addiction with Johnson-Neyman confidence bands


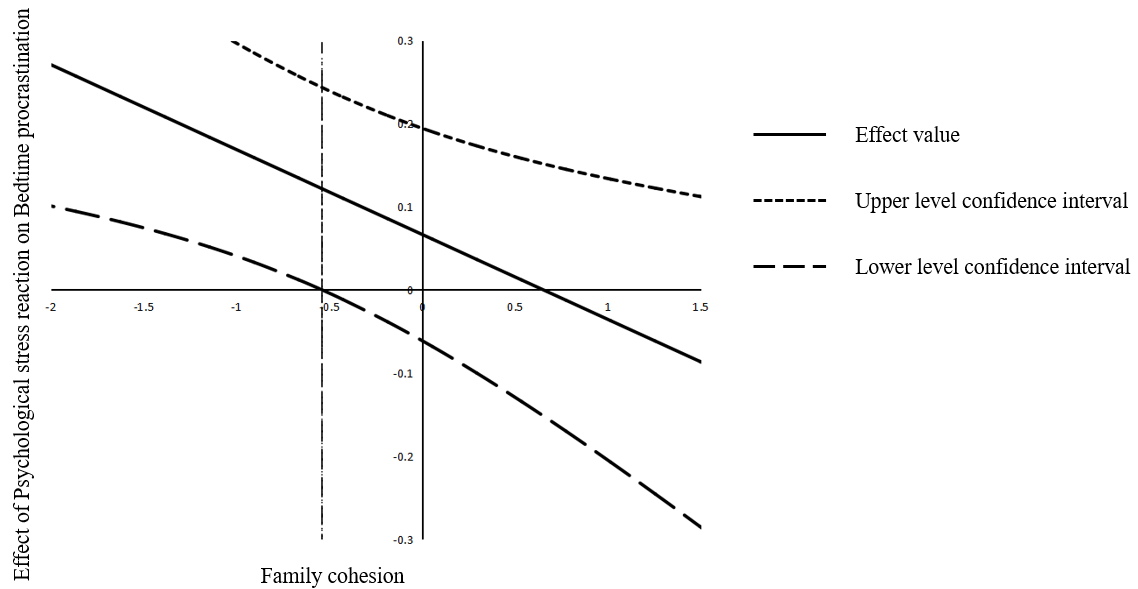


**Figure S2.** Effect of Psychological stress reaction on Bedtime procrastination with Johnson-Neyman confidence bands


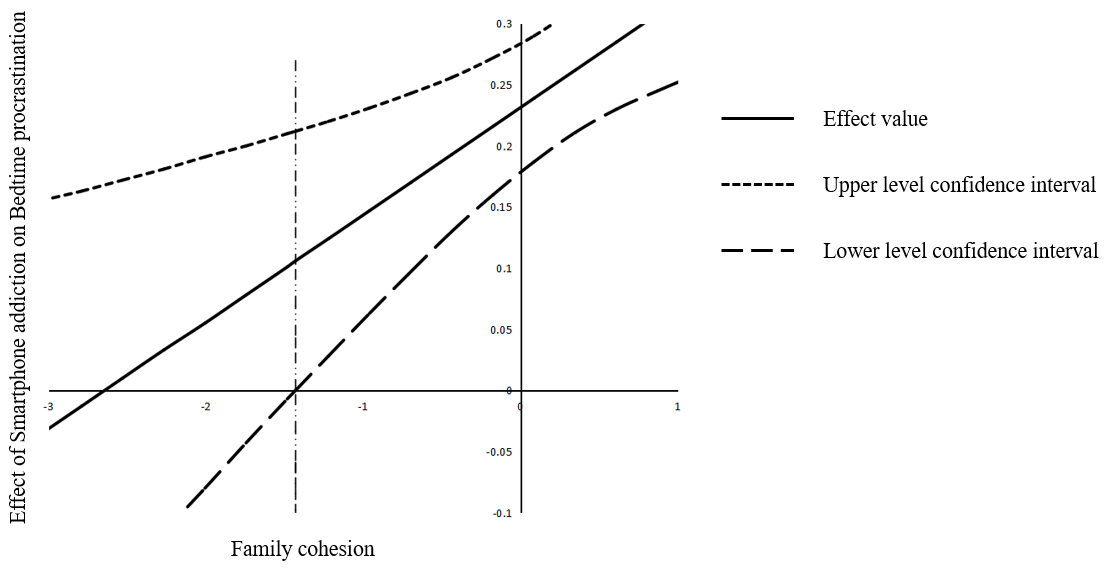


**Figure S3.** Effect of Smartphone addiction on Bedtime procrastination with Johnson-Neyman confidence bands
